# Endothelial progenitor cells as biomarkers of diabetes-related cardiovascular complications

**DOI:** 10.1186/s13287-023-03537-8

**Published:** 2023-11-10

**Authors:** Josefa Benítez-Camacho, Antonio Ballesteros, Lucía Beltrán-Camacho, Marta Rojas-Torres, Antonio Rosal-Vela, Margarita Jimenez-Palomares, Ismael Sanchez-Gomar, Mª Carmen Durán-Ruiz

**Affiliations:** 1 Biomedicine, Biotechnology and Public Health Department, Science Faculty, Cádiz University, Torre Sur. Avda. República Saharaui S/N, Polígono Río San Pedro, Puerto Real, 11519 Cádiz, Spain; 2 Biomedical Research and Innovation Institute of Cadiz (INIBICA), Cádiz, Spain; 3 Maimonides Biomedical Research Institute of Cordoba (IMIBIC), Córdoba, Spain; 4 Cell Biology, Physiology and Immunology Department, Córdoba University, Córdoba, Spain

**Keywords:** Diabetes mellitus, Diabetic vascular complications, Endothelial progenitor cells, Biomarkers

## Abstract

Diabetes mellitus (DM) constitutes a chronic metabolic disease characterized by elevated levels of blood glucose which can also lead to the so-called diabetic vascular complications (DVCs), responsible for most of the morbidity, hospitalizations and death registered in these patients. Currently, different approaches to prevent or reduce DM and its DVCs have focused on reducing blood sugar levels, cholesterol management or even changes in lifestyle habits. However, even the strictest glycaemic control strategies are not always sufficient to prevent the development of DVCs, which reflects the need to identify reliable biomarkers capable of predicting further vascular complications in diabetic patients. Endothelial progenitor cells (EPCs), widely known for their potential applications in cell therapy due to their regenerative properties, may be used as differential markers in DVCs, considering that the number and functionality of these cells are affected under the pathological environments related to DM. Besides, drugs commonly used with DM patients may influence the level or behaviour of EPCs as a pleiotropic effect that could finally be decisive in the prognosis of the disease. In the current review, we have analysed the relationship between diabetes and DVCs, focusing on the potential use of EPCs as biomarkers of diabetes progression towards the development of major vascular complications. Moreover, the effects of different drugs on the number and function of EPCs have been also addressed.

**Graphical Abstract:**

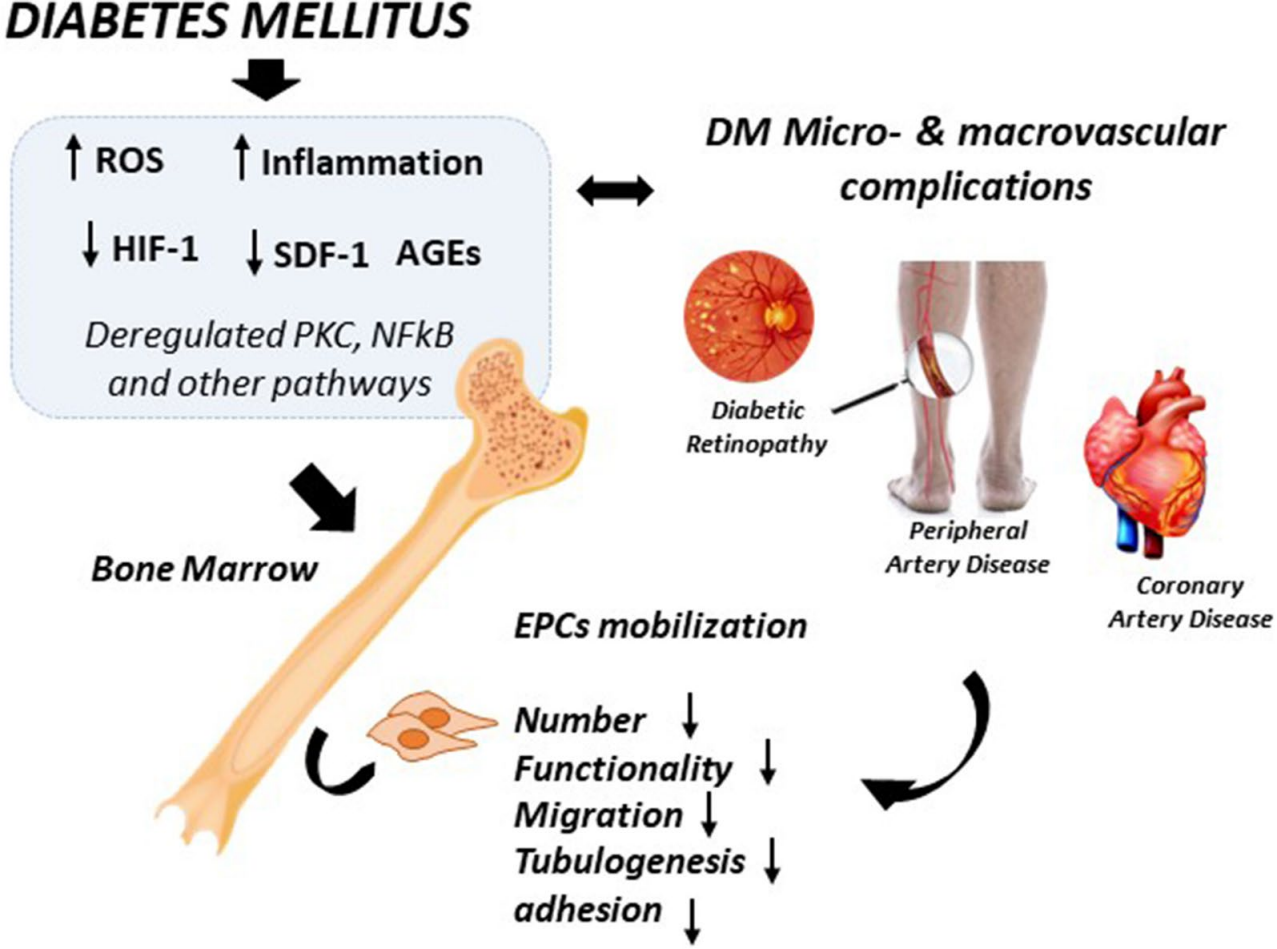

## Diabetes mellitus

Diabetes mellitus (DM) is considered a chronic and multifactorial metabolic disorder characterized by elevated levels of blood glucose which result from a lack in insulin secretion, insulin action or both, and it is associated with serious damage and dysfunction overtime of the eyes, kidneys, nerves, heart, and blood vessels between other organs [1]. According to the International Diabetes Federation, approximately 537 million people worldwide suffered from DM in 2021, being this disease the cause of 6.7 million deaths in the same year; furthermore, the number of diabetic people is expected to increase to 643 million by 2030 and 783 million by 2045 [2].

Diabetes can be classified in different groups [3]. Firstly, gestational diabetes mellitus (GDM) is understood as a temporary phase of glucose intolerance that affects 25% of pregnant women, and both, affected women and their off-springs, are known to deal with a higher risk of developing type 2 diabetes mellitus (T2DM) [4, 5]. Secondly, type 1 diabetes mellitus (T1DM) is caused by insulin deficiency due to an autoimmune-mediated destruction of pancreatic *β*-cells, which leads to an enhancement of hyperglycaemia and oxidative stress, alterations in lipid metabolism, and it is also related to endothelial cell dysfunction and apoptosis [6]. In contrast, T2DM takes place when the body cannot respond fully to insulin, followed by an increase in insulin production and subsequent insulin deficiency, heading to a permanent hyperglycaemia and glucose intolerance [7]. Remarkably, T2DM accounts for more than 90% of all diabetic people, exerting a wider contribution to the rising prevalence of DM globally compared to T1DM [8].

Although genetic predisposition plays an important role in T2DM, other factors such as age, gender, ethnicity, high body mass index, visceral obesity, high sugar intake, GDM, hypertension and dyslipidaemia, constitute the main non-genetic risk factors [9–11]. T2DM is closely related to obesity and insulin resistance (IR) [12, 13]. The last one, IR, begins years before T2DM in skeletal muscle, adipose tissue and liver, and it is thought to be primarily caused by ectopic lipid accumulation, as well as other factors like endoplasmic reticulum stress and inflammation [14–16]. Likewise, obesity is a IR risk factor due to the presence of fatty acids and inflammatory cytokines and therefore usually drives towards T2DM [17]. At first, pancreatic β-cells tend to increase the production of insulin in response of IR to overcome tissue requirements; however, β-cells number and functionality decline over time, and insulin secretion is insufficient, leading to the development of T2DM [18, 19]. β-cells impairment is mainly related to lipotoxicity, glucotoxicity and glucolipotoxicity. Chronic exposure to saturated fat and hyperglycaemia leads to metabolic, oxidative, and inflammatory stress that cause *β*-cell exhaustion, apoptosis, and loss of *β*-cells mass [18, 20, 21]. T2DM is preceded by an asymptomatic phase of prediabetes characterized by compromised glucose metabolism without fulfilling the criteria to be classified as diabetes [22]. This state has also been associated with the complications of diabetes, although it can be reversed through physical exercise and weight loss [18, 22]. The main biomarker used for the identification of diabetes and prediabetes is glycosylated haemoglobin (HbA1c), which correlates with chronic hyperglycaemia [23]. Although some patients are initially asymptomatic, polyuria, polydipsia, weight loss, polyphagia, and blurred vision can be also indicators of diabetes [18].

## Diabetes mellitus and cardiovascular complications

The connection between DM and cardiovascular diseases (CVD) has been related to how IR and hyperglycaemia promote atherosclerosis through several pathways, including chronic inflammation and oxidative stress [24, 25]. Atherosclerosis is a complex process involving different cell types and cell-to-cell interactions, characterized by focal deposits of cholesterol and lipids in the arterial intima, named atherosclerotic plaques. Hyperglycaemia, oxidative stress, and systemic inflammation lead to the damage of endothelial cells (EC) and inflammation of the artery intimal layer [26]. Immune circulating cells adhere to the injured area and then penetrate and differentiate into macrophages that participate in lipid uptake and accumulation of foam cells [25]. Subsequent plaque formation causes the narrowing of blood vessels lumen, which can progress towards thrombotic events and further complications such as coronary artery disease (CAD), heart failure, cerebrovascular disease, or peripheral artery disease (PAD) [27]. Therefore, current strategies to beat DM are not only focused on modulating hyperglycaemic levels, but also to modulate pro-atherosclerotic factors such as dyslipidemia, hypertension, or by application of antithrombotic therapies [28].

The risk of CVDs in adults with diabetes is two- to four-times greater than non-diabetic patients, representing nowadays a major burden of healthcare expenditure [29–32]. Overall, DM greatly increases the risk of vascular complications, the so-called diabetic vascular complications (DVCs), responsible for most of the morbidity, hospitalizations and death registered in these patients [29, 33, 34]. Thus, chronic hyperglycaemia induces pathognomonic changes resulting in ‘microvascular´ (affecting small blood vessels) and ‘macrovascular complications’ (due to damage to the arteries) [35]. Microvascular complications include diabetic retinopathy (DR), diabetic nephropathy (DN) and neural damage or diabetic neuropathy (DNeu). Macrovascular complications include CAD, the major responsible of mortality in diabetic patients, and PAD or the most severe form of it, critical limb ischemia (CLI), as the principal cause of lower extremity amputations [36–38]. There is not much clarity on whether microvascular complications precede macrovascular ones, or whether they progress simultaneously or independently. DM induces changes in the microvasculature, causing extracellular matrix protein synthesis, and thickening of capillary basement membrane. All this, in conjunction with advanced glycation end products, oxidative stress, low grade inflammation, and neovascularization of vasa vasorum, can lead to macrovascular complications [39].

Regarding microvascular complications, DNeu constitutes the most common prevalent DVC, affecting over 50% of diabetic patients [40, 41], and this prevalence increases with disease duration [42]. Clinically, DNeu preferentially targets sensory and autonomic axons and, in most severe cases, motor axons, and is headed as diffuse or focal neuropathies, depending on the location of nerve fibres involved [43]. On the other hand, DN occurs in 20–50% of diabetic patients, and it constitutes the major risk for end-stage kidney disease [44]. DN constitutes a highly complex process, which involves changes in renal structure and function [45], promoting an increase of albumin excretion and impaired glomerular filtration rate, among others [35, 46].

Finally, DR represents the leading cause of blindness and vision loss worldwide [47], with an annual estimated prevalence ranging from 2.2 to 12.7% [48]. DR is caused by changes in vascular permeability, capillary microaneurysms and degeneration, excessive formation of new blood vessels and impairment of the neural retina [49]. DR can be divided into non-proliferative (NPDR) and proliferative DR (PDR) [50]. In non-proliferative initial stages, patients do not suffer from visual impairments; however, hyperglycaemia produces loss of intramural pericyte and the progressive thickening of the basement membrane, altering vascular permeability and the blood-retinal barrier [35]. In more severe stages, capillary degeneration or occlusion generates an ischemia that promotes the release of angiogenic factors. The formation of new blood vessels and accumulation of liquid in the retina (diabetic macular oedema) triggers the progression into a proliferative stage, in which patients suffer from visual impairments [51].

Regarding macrovascular complications, DM is considered a risk factor in the development of CAD, one of the main macrovascular complications of T2DM [52, 53]. Indeed, CAD morbidity and mortality rates are higher in the presence of diabetes [54]. Besides, after suffering a myocardial infarction, diabetic patients are more likely to suffer re-infarction or to die than non-diabetic ones [55]. Similarly, DM is also a major risk factor in the development of PAD, having diabetic patients two-to seven-fold increased prevalence of PAD compared to non-diabetic population [56]. Currently, PAD is considered the most prominent form of atherosclerosis, which affects 5–10% of the adult population (> 202 billion people worldwide) [57]. The pathophysiology consists on the arterial obstruction and decrease of blood flow and therefore and reduction of oxygen and nutrients supply to the lower extremities [58]. In the presence of DM, hyperglycaemia correlates with platelet aggregation, inflammation, endothelial dysfunction and vascular smooth muscle cell dysfunction, mechanisms that promote the formation of atheromatous plaques that block arteries supplying blood to the extremities in the pathophysiology of diabetic PAD [59]. Furthermore, DM accelerates the progression of PAD to CLI [60], which courses, among others, with ischemic ulcers and gangrene of the extremities [61]. The risk of amputation is 7–15 times higher in diabetic CLI that in non-diabetic CLI [62], negatively affecting the quality of life of these patients, but it also increases the mortality rate [63].

Finally, the presence of comorbidities in patients with micro- or macrovascular diabetic complications is frequent. For instance, several studies indicate that DR, particularly PDR, and PAD are closely related, as assessed by Ankle Brachial Index, Toe Brachial Index and duplex ultrasonography [64]. PDR and PAD share several risk factors such as hyperglycaemia, blood pressure, dyslipidemia or albuminuria [64], and in both cases, neovascularization/angiogenesis is involved [65]. PDR (also related to atherosclerosis) is a strong risk factor for PAD [64, 66, 67], being PDR patients more likely to suffer PAD than NPDR patients, and moreover, PDR-T2DM patients have a high prevalence of PAD. In addition, DR appears to be a key factor for lower extremity amputation in patients with T1DM and T2DM [68]. Likewise, T2DM patients who undergo lower extremity amputation (LEA) present higher risk of developing DR than those without LEA [69]. In addition, some diabetic patients seem to develop severe DR and PAD earlier and more aggressive than others, independently of glycaemic control and measured environmental factors [70, 71].

## Endothelial progenitor cells

Since their discovery, endothelial progenitor cells (EPCs) have been defined as bone marrow (BM)-derived cells that are mobilized into the bloodstream after being stimulated endogenously or exogenously in response to different pathological processes such as atherosclerosis or ischemic damage [72, 73]. Once into the circulation, EPCs help to promote the restoration of the damaged endothelium [74, 75]. EPCs form a heterogeneous population that may differ in origin (BM, spleen, vascular endothelium, adventitia) and can growth in adherence to matrix molecules like fibronectin. EPCs were initially characterized by the uptake of 1,1‐dioctadecyl‐3,3,3,3‐tetramethylindocarbocyanine‐labelled acetylated low‐density lipoprotein (Dil‐acLDL) and the binding of fluorescein‐isothiocyanate (FITC)‐conjugated Ulex europaeus agglutinin lectin (FITC‐UEA‐I) [76]. Currently, several surface markers have also been associated with these cells, including endothelial (vascular endothelial growth factor receptor 2, VEGFR-2 or KDR; CD31 and von Willebrand factor, vWF) and hematopoietic markers (CD34, CD133 and CD45) [74, 75, 77]. EPCs can be obtained from different sources like BM, umbilical cord blood, adipose tissue and peripheral blood (PB) [78].

EPCs are currently classified in two main populations: early EPCs (eEPCs), also known as circulating angiogenic cells (CACs) or myeloid angiogenic cells, and late outgrowth EPCs or endothelial colony-forming cells (ECFCs) [79–82]. eEPCs are considered as angiogenic cells with myeloid features [80, 83], characterized by a spindle shape and the expression of several cell surface markers like CD45, CD14, CD31 CD133, CD34 and KDR. Moreover, eEPCs have a low proliferative potential and they cannot form colony or meshes in culture during in vitro assays [80, 83]. eEPCs participate in revascularization mainly through a paracrine manner, by the secretion of several factors such as VEGF, IL2, IL8, G-CSF, GM-CSF, HGF, or IL10, to promote the activity of ECFCs and other cells [84]. In addition, it has been described that eEPCs can produce apoptotic bodies, microvesicles and exosomes to stablish communication [84]. In response of atherosclerotic, ischemic, angiogenic or growth factors, eEPCs are chemoattracted to the affected area and become activated [73, 85, 86], promoting the regeneration of the damaged endothelium. Although the molecular mechanisms that support the paracrine action of eEPCs are not completely understood, the result of its secretory activity is the creation of an angiogenic microenvironment that boost the reparation of the endothelium and the revascularization process [87].

The second main cell type is ECFCs, which are recognized as cobblestoned cells with self-renewal potential [88] and membrane markers resembling those of EC, being positive for CD31, CD34, CD105, CD146, and negative for CD45 and CD14 [83, 89]. Sometimes, ECFCs have also been reported to express low levels of CD45 (CD45^dim^) [90] and high levels of CD34 and KDR [91]. ECFCs can be isolated from peripheral blood mononuclear cells (PBMCs), umbilical cord blood, adipose tissue or from tissue-resident vascular endothelium [80]. ECFCs display a great proliferative ability and possess a strong angiogenic potential, enabling the formation of tube-like structures in vitro. Compared to eEPCs, ECFCs are considered the “true” EPCs, due to their participation in revascularization by direct incorporation in the newly formed vessels in vivo [92]. Aside, a third subpopulation of EPCs, called colony-forming units (CFU)-Hill cells, derived from the culture of non-adherent PBMC has also been proposed [93]. CFU-Hill cells are formed by a heterogeneous group of KDR and CD31 positive cells and form colonies that include round cells at the centre surrounded by spindle-shaped cells [91].

### EPCs as biomarkers of cardiovascular risk

**Fig. 1:**
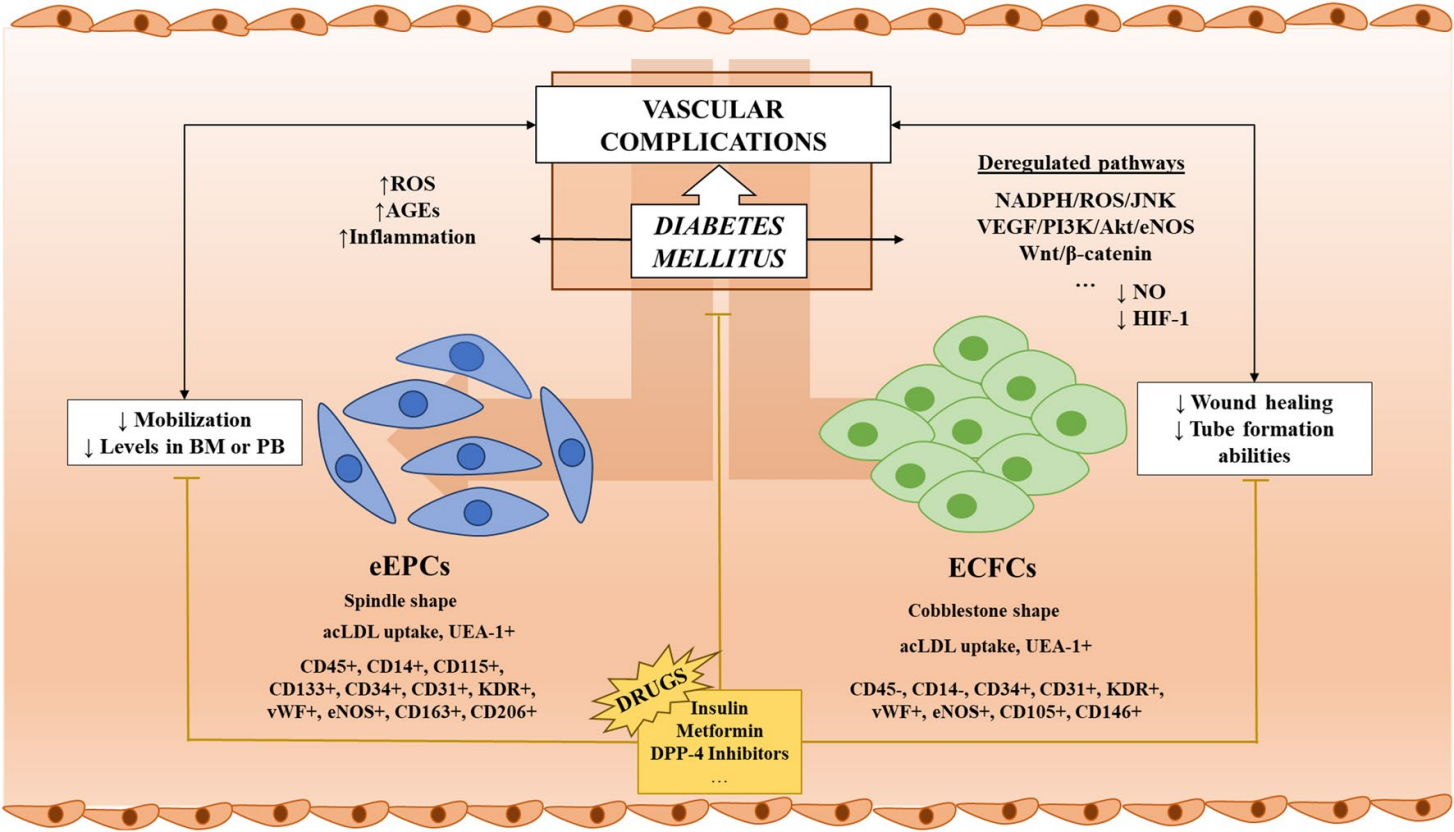
EPCs in diabetes mellitus and vascular complications. In the presence of DM and hyperglycaemia, there is an increment of inflammation and ROS and AGEs generation, which are associated with the deregulation of important biochemical pathways (NADPH/ROS/JNK, VEGF/PI3K/Akt/eNOS, Wnt/β-catenin…) affecting EPCs performance: eEPCs mobilization is negatively affected by these conditions, leading to a reduction in eEPCs number in BM and PB. Meanwhile, ECFCs functionality (wound healing and tube formation abilities, angiogenesis, migration, proliferation…) is compromised due to the hostile diabetic environment. All these abnormalities promote the development of DVCs (PAD, DR, DNeu, DN…). Further, DVCs aggravate the pathological environment, damaging EPCs behaviour

As reported above, EPCs play an important role in the maintenance of the cardiovascular system by participating in the healing of the damaged endothelium and in the neovascularization process [94]. However, in the presence of cardiovascular risk factors like metabolic syndrome, hypercholesterolemia, hypertension chronic kidney disease, smoking, and diabetes, the number and functionality of EPCs are negatively affected, causing a detrimental effect on the conservation of the healthy endothelium and the consequent worsening of endothelial dysfunction, atherosclerosis and cardiovascular disease [95]. In this regard, EPCs are currently considered as potential biomarkers of vascular homeostasis and cardiovascular risk prognosis, due to the reduced number and impaired function in the presence of DM (Fig. 1), although a better understanding of their implications in these pathologies is needed [96]. Different studies have analysed the connections between EPCs and diabetes and their use as potential biomarkers of DVC, evaluating the effect of DM and DVCs over eEPCs or ECFCs number/functionality. However, most articles do not totally clarify the subpopulation of EPCs studied. Thus, in order to facilitate the comparison between different studies, the EPCs phenotypes based on the cell surface markers used for cell identification, have been highlighted in the present review.

### EPCs in diabetes mellitus

To date, many studies have evaluated the levels of EPCs in DM (Table 1), finding similar results. In terms of T1DM, authors like Maiorino et al. and Salem et al. have stated that the low number of circulating EPCs (cEPCs: CD34^+^CD133^+^KDR^+^) could be considered as a predictor of the cardiovascular risk and the mortality income of these patients, existing an inverse correlation between glucose levels and cEPCs number [97, 98]. Moreover, T1DM has been recently associated with a worsened EPCs mobilization after exercising and reduced EPCs number in PB [99, 100]. Regarding T2DM, the levels of circulating PB cells (CPCs: CD34^+^) and EPCs (CD34^+^ KDR^+^) appear to be reduced in T2DM patients [101]. Also, cEPCs (CD34^+^/CD34^+^ KDR^+^) and CACs (KDR^+^ eNOS^+^ collagen type 1 Col1+) levels were found reduced in T2DM patients, while smooth muscle progenitor cells (SMPCs: CD14^+^ CD105^+^) levels did not change, causing an imbalance in the cEPCs/CACs-SMPCs ratios in these patients [102]. Besides, the lower numbers of EPCs (CD34^+^KDR^+^) appeared inversely correlated with plasma glucose and HbA1c levels in T2DM patients [103]. Other researchers reported an increase of cEPCs levels (CD34^+^CD133^+^KDR^+^) after two months of glycaemic control in newly-diagnosed diabetic patients, T1DM patients and diabetic patients without complications, while the levels did not change in patients with pre-existing DM, T2DM patients, and diabetic patients with DVC [104]. Furthermore, Egan et al. claimed that the low levels of the called “putative EPCs” (pEPCs; CD34^+^/CD31^+^CD34^+^/CD117^+^CD34^+^/CD133^+^CD34^+^/KDR^+^CD34^+^), in addition to the levels of haematopoietic cells (KDR^+^CD117^+^/CD133^+^ KDR^+^), could be used as a biomarker of the mortality risk over a period of 10 years in T2DM patients [105, 106].

**Table 1:** Clinical studies analysing EPCs levels in DM patients

Author (year)	DM	EPCs phenotypes	EPCs number in DM versus HC	References
Maiorino, M.I. (2015)	T1DM	cEPCs: CD34^+^ cEPCs: CD133^+^ cEPCs: CD34^+^ CD133^+^ cEPCs: CD133^+^ KDR^+^ cEPCs: CD34^+^ CD133^+^ KDR^+^	↓T1DM	[97]
Salem, M.A. (2022)	T1DM	cEPCs: CD34^+^ CD133^+^ KDR^+^	↓T1DM	[98]
Taylor, G.S. (2021)	T1DM	HPCs: CD34^+^ HPCs: CD34^+^ CD45^dim^ EPCs:CD34^+^ KDR^+^ EPCs: CD34^+^ CD45^dim^ KDR^+^	↓Mobilization after exercise	[99]
Maio, A. (2022)	T1DM	EPCs: CD34^+^ EPCs: CD34^+^ CD133^+^ EPCs: CD34^+^ KDR^+^	↓T1DM	[100]
Fadini, G.P. (2005)	T2DM	CPCs: CD34^+^ EPCs: CD34^+^ KDR^+^	↓T2DM	[101]
Van Ark, J. (2012)	T2DM	cEPCs: CD34^+^ cEPCs: CD34^+^ KDR^+^ CACs: KDR^+^ eNOS^+^ Col1^+^ SMPCs: CD14^+^ CD105^+^	↓T2DM ↓T2DM ↓T2DM NC T2DM	[102]
Churdchomjan, W. (2010)	T2DM	EPCs: CD34^+^ KDR^+^	↓T2DM	[103]
Bonora, B.M. (2022)	T1DM /T2DM	cEPCs: CD34^+^/CD133^+^KDR^+^	↑ Glycaemic control	[104]
Egan, C.G. (2008)	T2DM	pEPCs: CD34^+^ pEPCs: CD31^+^ CD34^+^ pEPCs: CD117^+^ CD34^+^ pEPCs: CD133^+^ CD34^+^ pEPCs: KDR^+^ CD34^+^ HC: KDR^+^ CD117^+^ HC: CD133^+^ KDR^+^	↓T2DM	[105]
Egan, C.G. (2018)	T2DM		↓T2DM	[106]
Fadini, G.P. (2013)	T2DM	CPCs: CD34^+^	↓T2DM (BM)	[110]

Different clinical studies evaluating the number of EPCs in the peripheral blood (PB) of diabetic patients are shown

*T1DM* type 1 diabetes mellitus, *T2DM* type 2 diabetes mellitus, *HPCs* hematopoietic progenitor cells, *EPCs* endothelial progenitor cells, *cEPCs* circulating endothelial progenitor cells, *CPCs* circulating peripheral blood progenitor cells, *CACs* circulating angiogenic cells, *SMPCs* smooth muscle progenitor cells, *pEPCs* putative endothelial progenitor cells, *HC* healthy control, *wo* without, *NC* no changes

The reduction in the number of EPCs in DM could be explained by both defects in the mobilization of EPCs from the BM and/or by pathological changes in the BM itself. Thus, the impaired mobilization of EPCs seen in DM has been linked to a deficiency in the enzymatic activity of the endothelial nitric oxide synthase (eNOS), essential for EPCs homing, which might be related to the diabetes-induced reduction of the lymphocyte and mesenchymal stromal populations in the BM [107]. Similarly, a reduced expression of nicotinamide phosphoribosyltransferase, an essential enzyme for NAD biosynthesis, in BM-derived cells in diabetic *db/db* mice, essential for NAD biosynthesis, was also associated with a lower mobilization of EPCs [108]. Meanwhile, the histopathology of diabetic BM has been reported, finding a diminished hematopoietic tissue, fat deposition, microvascular density reduction and apoptotic activation in the BM [109], as well as a decrease in CD34^+^ cells from BM aspirates of T2DM patients [110].

### Impaired functionality of ECPs under hyperglycaemic conditions

EPCs functionality is also affected in DM. Loomans et al. [111] reported that EPCs (Dil-acLDL^+^ FITC‐UEA‐I^+^) isolated from T1DM patients presented reduced angiogenic abilities, although apoptosis was not altered compared to EPCs isolated from healthy controls (HC). Besides, ECFCs from T2DM patients have been found to exhibit reduced proliferative and migratory abilities [112], and EPCs (Dil-acLDL^+^ FITC‐UEA‐I^+^ CD34^+^ CD31^+^CD146^+^KDR^+^) isolated from T2DM patients showed reduced proliferation, adhesion and tube forming ability [113]. Also, a lower isolation rate of EPCs and ECFCs from the PB of T2DM patients in comparison with HC has been described [114]. Similarly, CD34^+^ cells from T2DM patients exhibited a reduction in its vasodilatory, proliferative, migratory and angiogenic function, that seemed to be associated with changes in its secretory profile and a worsening of the response to hypoxia [114]. Also, the diminished angiogenic potential of ECFCs (Dil-acLDL^+^ FITC‐UEA‐I^+^ CD34^+^KDR^+^vWF^+^CD144^+^) from T2DM patients was associated with the impaired production of angiogenic cytokines [115]. Moreover, the in vitro high glucose exposure (HGE) of EPCs caused detrimental effects on viability, migration, proliferation and angiogenesis, with an increase in apoptosis in these cells [103, 116], while hyperinsulinemia triggered a reduction in the in vitro proliferation and tube formation ability of EPCs (Dil-acLDL^+^ FITC‐UEA‐I^+^ CD34^+^CD105^+^ CD106^+^CD133^+^VEGFR2^+^vWF^+^) and increased apoptosis by downregulation of the PI-3K/Akt/eNOS pathway and upregulation of p38 MAPK [117].

Concerning the mechanistic aspects that could explain the reduced number and dysfunction of diabetic EPCs, different factors like oxidative stress, glycation of lipid and proteins or the lower production of NO have been addressed [118–122]. The high presence of ROS has been associated with a reduction in the levels of the hypoxia inducible factor-1 (HIF-1), hampering the under-hypoxia expression of genes related to EPCs mobilization and angiogenesis, like SDF-1 and VEGF [119, 123]. Also, the presence of advanced glycation-end products (AGEs) in DM negatively affects EPCs functionality through the activation of RAGE and the affection of the NADPH/ROS/JNK pathway [124]. Likewise, the dysfunctionality of diabetic EPCs has been associated with a dysregulation of the VEGF/PI3K/Akt/eNOS pathway, mainly due to the reduction of the NO availability [125]. In accordance, defects in the production of NO by eNOS and a major production of superoxide anion (O_2_^−^) were also related to the lower levels of EPCs found in DM patients and their impaired functionality [121]. Besides, the inhibition of the Wnt/β-catenin pathway has been also reported to dysregulate DM-EPCs and to inhibited wound healing [116, 126]. In addition, the platelet-derived growth factor (PDGF) signalling pathway seemed to be related to the dysfunctionality of diabetic EPCs, although more research is needed to better understand the underlying mechanisms [116, 127]. Recently, Tiang et al. [128] reported that the increased autophagy and apoptosis found in EPCs after incubation under HGE were caused by the activation of circ-ADAM9, a circRNA molecule which induces autophagy by activation of PTEN, through the AKT/mTOR pathway. Finally, a transcriptome analysis revealed that Rno-miR-10b-5p and Tgfb2 are important regulators of EPCs dysfunction in diabetes, proposing novel targets to recover EPCs functionality [129].

### EPCs in DVC

The connection of EPCs with cardiovascular risk conditions, as well as the correlation of diabetic impaired EPCs with the occurrence and severity of micro- and macrovascular complications, suggests their suitability as biomarker of DVC, as well as their potential use as predictor of CVD outcomes, which could be of particular interest for diabetic patients [130, 131].

### EPCs in peripheral artery disease and critical limb ischemia

There is a current controversy regarding the behaviour of EPCs in PAD and diabetic PAD. However, such dispute may be explained by the different methods applied for cell counting and a lack of agreement in the nomenclature used between researchers, which sometimes causes the inappropriate comparison of different EPCs subtypes [132] (Table 2).

**Table 2:** Studies analysing EPCs levels in DM patients with PAD and CLI

Author (year)	DM	EPCs phenotypes	EPCs number	Control	References
Bitterli, L (2016)	DM	cEPCs: CD34^+^ KDR^+^	↓PAD with and wo DM	HC	[133]
Delva, P (2008)	DM	cEPCs: CD34^+^ cEPCs: CD34^+^ CD133^+^ ECFCs: CD34^+^ CD31^+^ CD144^+^	↓PAD with and wo DM ↓PAD with and wo DM ↑PAD with and wo DM	HC	[132]
Fadini, GP (2006)	T2DM	cEPCs: CD34^+^ KDR^+^ cEPCs: CD133^+^ KDR^+^ cEPCs: CD34^+^CD133^+^ KDR^+^	↓DM-PAD	DM	[134]
Krutikov, A (2009)	DM	EPCs: CD34^+^ CD133^+^ EPCs: CD34^+^ CD133^+^ KDR^+^	↑DM-PAD	DM	[135]
Chen, MC (2009)	T2DM	cEPCs: CD133^+^ KDR-1^+^	↓DM	HC and DM-CLI	[136]
Hayek, SS (2016)	DM	PCs: CD34^+^ PCs: CD34^+^/KDR^+^	↓CAD and PAD ↓CAD and PAD	CAD	[137]
Spinetti, G (2013)	T2DM	PCs: CD34^+^ CD14^+^ CD45^dim^ KDR^+^ CXCR4^+^	↓DM and CLI-DM	HC	[109]

*DM* diabetes mellitus, *T2DM* type 2 diabetes mellitus, *cEPCs* circulating endothelial progenitor cells, *EPCs* endothelial progenitor cells, *PCs* circulating progenitor cells, *PAD* peripheral artery disease, *CAD* coronary artery disease, *CLI* critical limb ischemia, *DNeu* neuropathy,: healthy control

To begin with, Bitterli et al. demonstrated that the levels of cEPCs (CD34^+^ KDR^+^) were lower in PAD patients in comparison with HC, and the colony-forming ability of these cells was also reduced in the disease group. Surprisingly, the levels of cEPCs in DM patients with PAD (PAD-DM) were slightly higher, not-significantly, than in PAD patients without DM, although the concomitant DM seemed to aggravate the disease, being related to a narrowing in the vessel wall [133]. Similarly, Delva et al. [132] found that when comparing EPCs isolated from PAD patients and HC, the results varied depending on the EPC subtype, finding a reduction in cEPCs (CD34^+^, CD34^+^ CD133^+^) but an augmentation in ECFCs (CD34^+^ CD31^+^ CD144^+^) in PAD patients versus HC. Despite this, no differences were seen in EPCs levels in PAD patients with and without DM [132]. Besides, several subpopulations of cEPCs (based on the differential expression of CD34, CD133 and KDR) were reduced in PAD-DM patients compared to DM patients without PAD, specially CD34^+^ KDR^+^ cells, which negatively correlated with the severity of PAD in DM patients [101, 134]. In this case, EPC functionality was determined by testing the properties of ECFCs (Dil-acLDL^+^), finding a reduced adhesion ability of cells derived from PAD-DM patients [134]. Contrary, Krutikov et al. [135] found an augmentation of EPCs (CD34^+^ CD133^+^/CD34^+^ CD133^+^ KDR^+^) levels in PAD-DM patients as compared with DM patients without PAD, being similar to the levels found in HC. In agreement with this, Chen et al. described a reduction in the levels of cEPCs (CD133^+^ KDR-1^+^) in DM patients compared to DM-CLI patients and HC. These cEPCs levels correlated with plasma VEGF levels [136]. However, the migratory ability of isolated EPCs in response to VEGF was impaired in DM and DM-CLI patients versus HC [136]. Further, a large cohort-study demonstrated that the levels of circulating progenitor cells (PCs) (CD34^+^/CD34^+^ KDR^+^) could be used as predictors of the development of PAD in patients with known CAD, being DM a risk factor in the development of PAD in these patients [137]. Meanwhile, Spinetti et al. [109] observed a reduction in the PCs levels (CD34^+^ CD14^+^ CD45^dim^ KDR^+^ CXCR4^+^) of DM patients in BM and PB in comparison with HC, although no differences were seen between DM and CLI-DM patients.

### EPCs in diabetic retinopathy

As it can be deduced from above, in macrovascular complications, dysfunctional EPCs may impede the compensatory angiogenesis necessary to reduce the progression of the ischemic process. Nevertheless, in complications such as DR, an excessive microvasculature formation worsens the patient’s condition [138, 139]. Interestingly, retinal cells release neurotrophic factors under hypoxia, so that DM patients experience poor vessel growth in heart and limbs, but due to the retinal microenvironment, they experience increased angiogenesis in the presence of retinal complications [140]. In this sense, diabetic patients face a paradoxical situation in which both, lower and excessive numbers of EPCs are associated with DVC [141]. This situation highlights the importance of addressing how EPCs are affected under DR, not only to use them as potential biomarkers to predict the development of the microvascular complication, but also to better understand the disease.

Still, the literature reflects conflictive results: no changes, reduction, and augmentation of EPCs levels; they all have been associated with DR (Table 3). First, Torre et al. [142] found that although T1DM patients faced lower levels of ECFCs (CD45^dim^ CD34^+^CD144^+^) than HC, there were not differences in the levels of ECFCs or eEPCs (CD45^dim^ CD34^+^KDR^+^) between DR and T1DM patients without DR. Meanwhile, Fadini et al. [141] determined that CD34^+^ cEPCs were reduced in DR patients (without discriminating between PDR and NPDR) while CD34^+^KDR^+^ cEPCs levels were lower in DM patients with PAD, existing differences in the CD34^+^/CD34^+^KDR^+^ ratio between DR and PAD patients. Contrary, Lee et al. [143] demonstrated that cEPCs (CD34^+^) were increased in PDR and NPDR patients compared to HC or DM patients without DR.

**Table 3:** Studies analysing EPCs levels in DM patients with DR

Author (year)	DM	EPCs phenotypes	EPCs number	Control	References
Fadini, GP (2006)	T2DM	cEPCs: CD34^+^ cEPCs: CD34^+^KDR^+^	↓DR; NC in PAD ↓PAD; NC in DR;	DM	[141]
Torre, N (2015)	T1DM	eEPCs: CD45^dim^ CD34^+^ KDR^+^ ECFCs: CD45^dim^ CD34^+^ CD144^+^	NC in DR	T1DM	[142]
Lee, IG (2006)	T2DM	cEPCs: CD34^+^	↑PDR; NPDR	HC and DM	[143]
Brunner, S (2009)	T1DM	CPCs: CD34^+^ CD133^+^ EPCs: CD34^+^ CD133^+^ CD309^+^ ECFCs: CD34^+^ CD133^+^ CD309^+^ CD31^+^	↓NPDR; ↑PDR	HC	[144]
Liu, X (2010)	T2DM	cEPCs: CD34^+^ CD133^+^	↑Severe NPDR to PDR	HC	[140]
Tan, K (2010)	T1DM	ECFCs: CD34^+^ CD45^−^	↑PDR	HC	[146]
Huang, YC (2018)	T2DM	ECFCs: CD31^+^ KDR-2^+^ CD45^dim^ CD133^+^	↓PDR	NPDR	[147]
Lombardo, M (2012)	T2DM	pre-EPCs: CD34^+^ CD133^+^ CD117^+^ EPCs: CD34^+^ CD133^+^ KDR^+^ late EPCs: CD31^+^ KDR^+^ VE-cadherin ^+^ CECs: CD45^−^ CD31^+^ CD146^+^ activated CECs: CD106^+^	↑DR-PAD ↓DR-PAD ↓DR-PAD ↑DR-PAD ↑DR-PAD	HC	[148]
Zerbini, G (2012)	T1DM	CFU-Hill cells: CD45^+^ CD14 ^+^ ECFCs: CD34^+^ KDR^+^ CD133^−^ CD45^dim^	↑NPDR NC in NPDR	HC	[149]
Hu, L (2011)	T2DM	CPCs: CD34^+^ EPOR^+^ EPCs: CD34^+^ KDR^+^ EPOR^+^	↓NPDR ↓NPDR ↓PDR ↓PDR-DN	HC	[150]

*DM* diabetes mellitus, *T1DM* type 1 diabetes mellitus, *T2DM* type 2 diabetes mellitus, *cEPCs* circulating endothelial progenitor cells, *eEPCs* early endothelial progenitor cells, *ECFCs* endothelial colony progenitor cells, *EPCs* endothelial progenitor cells, *CECs* circulating endothelial cells, *DR* diabetic retinopathy, *NC* no changes, *PDR* proliferative diabetic retinopathy, *NPDR* non proliferative diabetic retinopathy, *PAD* peripheral artery disease, *DN* diabetic nephropathy, *HC* healthy control

In an attempt to clarify these discrepancies, different subtypes of EPCs at different stages of DR have been evaluated. Thus, according to Brunner et al., the levels of different populations of EPCs: CPCs (CD34^+^CD133^+^), the most primitives EPC subtype; eEPCs (CD34^+^CD133^+^CD309^+^) and ECFCs (CD34^+^CD133^+^CD309^+^CD31^+^) were all lower in T1DM patients with NPDR but augmented in PDR. These results suggest that, although these cells undergo a reduction in the first stages of DR, later, their levels increase with the progression and severity of the disease [144]. Similarly, an augmentation of cEPCs (CD34^+^CD133^+^) in DM patients with severe NPDR to PDR in comparison with HC, and an increment in the colony-forming ability of cells isolated from patients with severe DR was also described by Liu et al*.* [145]. Of note, Tan et al*.* [146] reported that, despite the augmentation of circulating ECFCs levels (CD34^+^CD45^−^) in PDR, these cells showed impaired migratory and tube-forming abilities, being unable to repair the damage endothelium. Finally, although the majority of studies have seen increased levels of EPCs in PDR, other authors identified lower levels of EPCs (CD31^+^KDR-2^+^CD45^dim^ CD133^+^) in PDR compared to NPDR [147]. Besides, differences in EPCs subtypes behaviour were also seen by Lombardo et al., who classified EPCs in pre-EPCs (CD34^+^CD133^+^CD117^+^), EPCs (CD34^+^CD133^+^KDR^+^) and late EPCs (CD31^+^KDR^+^, VE-cadherin^+^). These authors indicated that T2DM patients with and without macro- and microvascular complications (primary PAD and DR) did not significantly differ in pre-EPCs and EPCs levels compared to HC [148]. However, a subset of both groups of patients seemed to have increased and decreased levels of pre-EPCs and EPCs, respectively, while late EPCs were augmented in both groups compared to HC [148]. Oppositely, Zerbini et al. [149] found that ECFCs (CD45^dim^ CD34^+^ KDR^+^) did not vary significantly between T1DM patients with NPDR and HC, although the number of CD45^+^CD14^+^ CFU-Hill cells increased in NPDR compared to HC, when measured as the number of colonies formed in vitro by 10^6^ PBMCs. These CFU-Hill cells presented a lower expression of genes associated with apoptosis (CASP1, CASP2) and cell–matrix interactions (integrins, ITGAV, ITGB1 and urokinase plasminogen activator, PLAU), and a lower expression of the homing receptor CXCR4 for SDF-1, which could be associated with their impaired functionality [149]. In addition, a study evaluating the role of the erythropoietin (EPO) and its receptor (EPOR) in different stages of DR, found that CPCs (CD34^+^ EPOR^+^) were reduced in NPDR patients compared to HC while those levels did not significantly vary in PDR patients and DR patients with diabetic nephropathy (DR-DN) [150]. Besides, EPCs levels (CD34^+^KDR^+^EPOR^+^) were significantly reduced in all groups compared to HC, although a rebound in PDR and PDR-DN patients was seen [150].

### EPCs in other DVCs

EPCs levels have been evaluated in other DVCs (Table 4), including microvascular complications like DNeu or DN. Thus, DNeu T2DM patients present increased numbers of the different subpopulations of EPCs compared to T2DM patients without DNeu [151]. On the other hand, although DN has been mostly related to a reduction in the EPCs number [152], similar levels of EPCs (Dil-acLDL^+^ FITC‐UEA‐I^+^ CD34^+^) were found in T1DM patients with and without DN, where DN patients faced a higher CVD risk, due to the longer diabetes duration, poorer glycaemic control and higher very low-density lipoprotein (VLDL) and triglycerides [153]. However, these results might have been influenced by the use of statins, which are known to increase EPCs number [153]. Finally, Pala et al. [154] reported lower levels of cEPCs (CD34^+^) in DM patients with DN who had developed stage 5 chronic renal disease (CRD) compared to HC, DM patients and CRD patients without DM.

**Table 4:** Studies regarding EPCs levels in other DVCs

Author (year)	DM	DVC	EPCs phenotypes	EPCs number	Control	References
Eleftheriadou, I (2020)	T2DM	DNeu	EPCs: CD45^dim^ CD34^+^ EPCs: CD45^dim^ CD34^+^ CD309^+^ EPCs: CD45^dim^ CD34^+^ CD31^+^ EPCs: CD45^dim^ CD34^+^ CD309^+^ CD31^+^ EPCs: CD45^dim^ CD34^+^ CD309^+^ CD133^+^ EPCs: CD45^dim^ CD34^+^ CD309^+^ CD133^+^ CD31^+^	↑DNeu	T2DM	[151]
Reinhard, H (2011)	T1DM	DN	EPCs: Dil-acLDL^+^ FITC‐UEA‐I^+^ CD34^+^	NC in DN	T1DM	[153]
Makino, H (2009)	T2DM	DN	cEPCs: CD34^+^	↓DN	T2DM	[152]
Pala, C (2013)	DM	DN (CRD, stage5)	EPCs: CD34^+^	↓DM-CRD	HC, DM wo DN, CRD wo DM	[154]

*DM* diabetes mellitus, *T1DM* type 1 diabetes mellitus, *T2DM* type 2 diabetes mellitus, *DNeu* diabetic neuropathy, *AMI* acute myocardial infarction, *DN* diabetic nephropathy, *CRD* chronic renal disease, *EPCs* endothelial progenitor cells, *cEPCs* circulating endothelial progenitor cells, *HC* healthy control, *wo* without

Finally, in correlation with all these DVCs, the involvement of EPCs in the thrombotic events linked to DM has been also explored. Indeed, EPCs seem to have an antithrombotic function, promoting the recanalization of the thrombus and the neovascularization of the damage tissue [155], although DM seem to reduce the antifibrinolytic activity of EPCs [130]. Interestingly, DM was found associated with a higher risk of developing vascular thrombosis in haemodialysis patients, with a negative correlation between thrombosis and EPCs number (CD34^+^ KDR^+^) [156]. Also, the levels of cEPCs (CD133^+^KDR^+^/CD34^+^ KDR^+^) were reduced in DM and non-DM patients that suffered stent thrombosis in comparison with those who did not [157]. Of note, retinal microthromboses are usually present in DR [158], although the role of EPCs in this concern is not fully understood and more research is needed.

Overall, the potential use of EPCs as biomarkers of DVC seems clear. Regarding macrovascular complications, a decrease in the number of EPCs has been frequently described, which negatively correlates with the severity of the disease, although different tendencies, such as increases in the number of EPCs with the disease or no changes, have been also reported. Such variations of EPC levels could be due to several factors, as previously mentioned, including the lack of heterogeneity in the type of EPC analysed, differences in the measurement systems used, the population sample size or even the drug treatments applied to DM patients. In microvascular complications, especially in DR, the issue is even more complicated, since depending on the state of the disease (NPDR, PDR) and the concomitance of macrovascular complications, it can be observed both, augmentations, and diminutions in the number of EPCs. Nevertheless, in all cases, the functionality of EPCs is compromised in the presence of DVC, so the next steps should be focused on a better understand why these cells become so affected under hyperglycaemic conditions.

### Drugs modulating EPCs levels in DM patients

Given the negative impact of DM and DVCs over EPCs function and numbers, the use of drugs contributing to ameliorate such effects may be useful to prevent further vascular complications. Unfortunately, the treatments commonly used for DM patients usually affect EPCs levels, either by controlling glucose and lipid levels itself and therefore reducing EPCs number, or by specific mechanisms that are not always fully understood [159]. Therefore, these pleiotropic effects should be considered during clinical trials that evaluate disease’s effects on EPCs, given that it could influence the results. Table 5 reviews the main studies that have evaluated the effect of several drugs used with DM patients over EPCs.

**Table 5:** Drugs effects on EPCs in DM patients

Drug	Drug versus Control	Experimental design	Population	Cells phenotype	Drug effect on EPCs	Author (Year)	References
Insulin	Insulin glargine versus insulin detemir (dose adjusted)	Randomized cross-over study (6 months)	T2DM (n:42)	cEPCs: CD133^+^ KDR^+^/CD34^+^ CD133^+^ KDR^+^	↑Number	Fadini, G.P. (2011)	[160]
	Insulin glargine and NPH insulin (dose adjusted) versus oral medication	Partially double-blind, randomized, three-arm unicentre study (4 weeks)	T2DM (n:55)	EPCs: CD34^+^ KDR^+^	↑Number	Oikonomou, D. (2014)	[161]
	Insulin pump versus multiple daily insulin injections	Observational (2 years)	T1DM (n:204)	CPCs: CD34^+^/CD133^+^/CD34^+^ CD133^+^ cEPCs: CD34^+^ KDR^+^/CD133^+^ KDR^+^/CD34^+^ CD133^+^ KDR^+^	↑Number	Longo, M. (2020)	[162]
	Continuous subcutaneous insulin infusion versus multiple daily injections (dose adjusted)	Open controlled study (6 months)	T1DM (n:106)	EPCs: CD34^+^ KDR^+^/CD34^+^ KDR^+^ CD133^+^	↑Number	Maiorino, M.I. (2016)	[163]
	Intensive insulin therapy (dose adjusted)	Open controlled study	T2DM (n:36)	EPCs: CD34^+^ KDR^+^ CD133^+^	↓Number	Zhang, W. (2022)	[164]
Sulfonylurea	Gliclazide (30–90 mg/day)	Open controlled study (12 weeks)	T2DM (n:33)	EPCs: CD45^dim^ CD34^+^ KDR^+^	↑Number	Chen, L-L. (2011)	[165]
Metformin	Metformin (500 mg OD–1 g BID)	Open label and parallel standard treatment study	T1DM (n:23)	cEPCs: CD45d^im^ CD34^+^ KDR^+^ cECs: CD45^dim^ CD133^−^ CD34^+^ CD144^+^ KDR^+^	↑Number ↓Number	Ahmed, F.W. (2016)	[169]
	Metformin (250–1000 mg/day) + Gliclazide (30–60 mg/day) versus Metformin (500–2500 mg/day)	Randomized open study (16 weeks)	T2DM (n:47)	cEPCs: CD45^dim^ CD34^+^ KDR^+^	↑Number	Chen, L.L. (2010)	[170]
	Insulin + metformin versus Metformin monotreatment	Open controlled study	T2DM (n:95)	cEPCs: CD34^+^ CD133^+^ KDR^+^	↑Number	Asadian, s. (2019)	[171]
Thiazolidinediones	Rosiglitazone (4 mg BID)	Open controlled study (12 weeks)	T2DM (n:10)	EPCs: CD34^+^/CD34^+^ CD133^+^	↑Number ↑Migration	Pistrosch, F. (2005)	[172]
	Rosiglitazone (4 mg BID)	Open placebo-controlled study (2 weeks)	T2DM (n:30)	EPCs: Dil-acLDL^+^ UEA-1^+^ CD31^+^ vWF^+^ KDR^+^ CD14^+^	↑Revascularization	Sorrentino, S.A. (2007)	[174]
	Pioglitazone (30 mg)	Randomized controlled study (8 weeks)	T2DM patients on metformin monotherapy (n:36)	EPCs: Dil-acLDL^+^ UEA-1^+^ vWF + Tie-2^+^	↑Number ↑Functionality	Wang, C.H. (2006)	[176]
DPP-4 inhibitors	Sitagliptin (100 mg/day)	Open controlled study (4 weeks)	T2DM (n:32)	EPCs: CD34^+^/CD34^+^ KDR^+^	↑Number	Fadini, G.P. (2010)	[179]
	Sitagliptin (50 mg/day) versus Placebo (Glimepiride,1 mg/day)	Prospective, randomized, open label clinical trial (12 weeks)	T2DM (n:30)	EPCs: CD34^+^ CXCR4^+^	↑Number	Aso, Y. (2015)	[180]
	Sitagliptin and metformin combined therapy versus monotherapies	Randomized controlled clinical trial (3 days)	T2DM (n:60)	EPCs: CD34^+^ KDR^+^/CD34^+^CD133^+^KDR^+^	↑Number	Xu, M. (2014)	[182]
	Sitagliptin (50 mg/day) versus Voglibose (0.6 mg/day)	Randomized prospective multicentre study (12 weeks)	T2DM (n:66)	EPCs: CD34^+^	↑Number	Nakamura, K. (2014)	[183]
	Vildagliptin (100 mg/day) versus Glibenclamide (2.5–5 mg BID)	Randomized open-label trial (12 months)	T2DM (n:64)	EPCs: CD34^+^ CD133^+^ KDR^+^	↑Number	Dei Cas, A. (2017)	[166]
	Alogliptin (25 mg/day) versus Gliclazide (30 mg/day)	Randomized clinical trial (4 months)	T2DM (n:80)	EPCs: CD133^+^ KDR^+^ CD45^−^/CD34^+^ KDR^+^/CD45^−^	↑Number	Negro, R. (2019)	[184]
	Saxagliptin (5 mg/day) versus Metformin (1500 mg/day)	Controlled, randomized, open-label clinical trial (12 weeks)	T2DM (n:27)	EPCs: CD34^+^ CD133^+^ KDR^+^	↑Number	Li, F. (2017)	[185]
	Saxagliptin (5 mg/day) and metformin (1–2 g/day) versus Metformin (1–2 g/day)	Phase 4, single-site, double-blind, placebo-controlled, randomized clinical trial (12 weeks)	T2DM (n:42)	cEPCs: CD34^+^/CD34^+^ CXCR4^+^ cECs: CD31^+^	↑ Proportion ↓Number	Dore, F.J. (2018)	[186]
	Linagliptin (5 mg/day)	Randomized, Placebo-controlled trial (12 weeks)	T2DM (n:40)	EPCs: 20 subpopulations based on CD34, CD133, KDR, and CD45 expression	NC	Baltzis, D. (2016)	[188]
	Linagliptin (5 mg/day)	Subanalysis from a randomized, placebo-controlled trial (RELEASE study) (26 weeks)	T2DM (n:41)	EPCs: CD34^+^ CD133^+^ KDR^+^ Tang cells: CD3^+^ CD31^+^ CXCR4^+^	NC ↑Number	de Boer, S.A. (2020)	[189]
	Teneligliptin (20 mg/day)	Single-centre, open-label, prospective, randomized controlled trial (28 weeks)	T2DM (n:17)	EPCs: CD34^+^	↑Number ↓ SDF-1α	Akashi, N. (2023)	[190]
GLP-1 Receptor Agonists	Exenatide (5 μg BID-10 μg TID) versus Liraglutide (0.6 mg OD-1.2 mg OD)	Comparative study (4–7 weeks)	T2DM (n:11)	EPCs: CD34^+^ KDR^+^	↑Number	De Ciuceis C. (2018)	[191]
	Liraglutide (1.2 mg/day)	Randomized open label trial (4 weeks)	T2DM (n:49)	HPCs: CD34^+^ CD45^dim^ HPCs with angiogenic activity: CD34^+^ CD45^dim^ CD133^+^ CD31^+^	NC	Gaborit, B. (2019)	[192]
	Liraglutide (1.8 mg OD) versus Sitagliptin (100 mg OD)	Randomized, active-comparator trial (26 week)	T2DM and obesity (n:61)	CPCs: CD34^+^/CD34^+^ CD133^+^/CD34^+^ CD45^dim^/CD34^+^ CD45^dim^ CD133^+^ EPCs: KDR coexpression	NC	Ahmad, E. (2021)	[193]
	Dulaglutide (1.5 mg/day) and metformin (1000 mg/day) versus Metformin (2000 mg/day)	Randomized controlled trial (12 weeks)	T2DM (n:60)	EPCs: CD34^+^ CD133^+^ KDR^+^	↑Number ↑Functionality	Xie, D. (2022)	[195]
SGLT2 inhibitors	Canagliflozin (100 mg OD)	Phase 4 (post-marketing), two arm, single site, parallel group, double blind, placebo controlled randomized clinical trial (16 weeks)	T2DM (n:29)	EPCs: CD34^+^	↑Functionality	Nandula, S.R. (2021)	[196]
	Dapagliflozin (10 mg) and empagliflozin (10 mg)	Randomized placebo open trial (Dapagliflozin, 12 weeks and 1.5 years, n:33) and open-label observational study (Empagliflozin, 12 weeks, n:15)	T2DM	CSC: CD34^+^ EPCs: CD34^+^ KDR^+^	↓Number ↓Number (12 weeks) ↑Number (1.5 years)	Bonora, B.M. (2018)	[197]
Statins	Atorvastatin (80 mg/day)	Open-labelled prospective trial (10 weeks)	DM (n:14) and non-DM (n:10) with CVDs	CPCs: CD45^−^ CD34^+^ CD133^+^	↑Number	Jaumdally, R.J. (2010)	[199]
	Pitavastatin (2 mg/day) versus Atorvastatin (10 mg/day)	Prospective, double-blind, randomized (12 weeks)	DM (n:19) and non-DM (n:7) with hyperlipidemia	EPCs: CD34^+^ KDR^+^	↑Number ↑Functionality	Lin, L.Y. (2014)	[200]
	Statins discontinuation	Randomized controlled trial (5 days)	T2DM (n:34)	EPCs: CD34^+^ KDR^+^/CD133^+^ KDR^+^/CD34^+^ CD133^+^ KDR^+^/CD34^+^ CD45^−^	↑Number	Fadini, G.P. (2015)	[201]
	High (80 mg/day)—or moderate (20 mg/say)—intensity atorvastatin therapy	Randomized trial (3 months)	DM with drug-eluting stent implantation (n:130)	EPCs: CD34^+^ KDR + 133^+^/CD34^+^ KDR^+^	↑Number	Briguori, C. (2017)	[202]
	Statins	Prospective observational study (3 months)	DM (n:62) and non-DM (n:38) with AMI and 2-years follow up period	EPCs: CD45^dim^ CD34^+^ KDR^+ ^CXCR4^+^/CD45^dim^ CD34^+^ KDR^+^ CD133^+^	↑Number	António, N. (2014)	[203]
	High-to-moderate intensity statin (atorvastatin 40–20 mg, fluvastatin 80 mg, simvastatin 80–20 mg) versus low intensity statin (fluvastatin 40–20 mg, pravastatin 20 mg, simvastatin 10 mg)	Monocentric prospective all-comers trial	DM (n:60) and non-DM (n:45) with coronary angiography	EPCs: CD34^+^ KDR^+^	↓Number	Florescu, R. (2022)	[204]
	Statins	Open labelled trial (3 months)	T2DM (n:45) and non-DM (n:37) at high cardiovascular risk	Total EPCs: CD45^−^ CD34^+^ eEPCs: CD45^−^ CD34^+^ CD146^−^	↓Number ↑PCSK9	Tripaldi, R. (2021)	[207]
PCSK9 inhibitors	Alirocumab (140 mg/ml twice a week) or evolocumab (75–150 mg/ml twice a week)	Open labelled trial (12 weeks)	DM (n:10) and non-DM (n:16) with PAD-CAD	cEPCs: CD34^+^ KDR^+^/CD133^+^ KDR^+^	↑Number ↑Functionality	Itzhaki Ben Zadok, O. (2022)	[206]
RAS inhibitors	Olmesartan (40 mg/day) versus Irbesartan (300 mg/day)	Double-blind placebo-controlled study (12 weeks)	T2DM (n:18)	EPCs: Dil-LDL^+^ UEA-1^+^	↑Number	Bahlmann, F.H. (2005)	[208]
Angiotensin-2 receptor blocker	Valsartan (adjusted dose)	Open study (52 weeks)	DM with asymptomatic CAD (n:86)	CD14^+^ KDR^+^	↑Number	Berezin, A.E. (2015)	[209]
Angiotensin-converting enzyme inhibitor	Perindopril (4 mg/day)	Open randomized controlled study (6 months)	T2DM with PCI (n:68)	cEPCs: CD34^+^ CD133^+^ KDR^+^	↑Number	Sun, J.Y. (2013)	[210]
Renin inhibitor	Aliskiren (150–300 mg/day) versus Hydrochlorothiazide (12.5-25 mg/day)	Open study (3 months)	T2DM with hypertension (n:20)	EPCs: acLDL^+^ UEA-1^+^	↑Number	Raptis, A.E. (2014)	[214]
P2Y12 receptor antagonists	Ticagrelor versus Prasugrel	Longitudinal study (AngioSafe 1) and randomized open label trial (AngioSafe 2) (10 weeks)	T2DM with non–ST-segment elevation acute coronary syndrome	EPCs: CD34^+^ KDR^+^/CD34^+^ CD117^+^/CD34 + CD133^+^	↑Number	Jeong, H.S. (2017)	[217]
Multifactorial treatment	Metformin, aspirin, statins and angiotensin II blockers	Open controlled study (90 days)	T2DM (n:28)	EPCs: Dil-acLDL^+^ UEA-1^+^	↑ Number	Reinhard, H. (2010)	[218]

*OD* once daily, *BID* bis in die, *TID* ter in die, *DM* diabetes mellitus, *T1DM* type 1 diabetes mellitus, *T2DM* type 2 diabetes mellitus, *PAD* peripheral artery disease, *CAD* coronary artery disease, *PCI* percutaneous coronary intervention, *HGE* high glucose exposure, *EPCs* endothelial progenitor cells, *cEPCs* circulating endothelial progenitor cells, *eEPCs* early endothelial progenitor cells, *CPCs* circulating progenitor cells, *HPCs* hematopoietic progenitor cells, *CSCs* circulating stem cells, *HSPCs* circulating haematopoietic stem, *NC* no changes, *PDR* proliferative diabetic retinopathy

### Insulin

The effect of insulin on EPCs has been widely studied. Thus, insulin treatment of streptozotocin (STZ)-induced DM mice increased EPCs mobilization (Dil-acLDL^+^ FITC‐UEA‐I^+^ Sca-1^+^ c-kit^+^ Flk-1^+^) and improved revascularization after hind limb ischemia (HLI), being this effect associated with the regulation of the VEGF/Akt/eNOS and SDF-1/MMP-9 pathway [159]. Likewise, clinical trials have shown similar results. Indeed, insulin therapy increased cEPCs levels (CD133^+^KDR^+^/CD34^+^CD133^+^KDR^+^) in T2DM patients after 6 months of treatment alternating basal insulin analogues glargine and detemir [160], and a 4-months-treatment of T2DM patients with either, insulin glargine or NPH insulin increased the outgrowth of EPCs (CD34^+^ KDR^+^) in comparison with oral medication [161]. Interestingly, a 2 years-treatment with insulin pumps increased the number of circulating progenitor cells (CPCs; CD34^+^/CD133^+^/CD34^+^CD133^+^) and cEPCs (CD34^+^KDR^+^/CD133^+^KDR^+^/CD34^+^CD133^+^KDR^+^) in T1DM patients, diminishing the cardiovascular risk [162]. Besides, a continuous subcutaneous insulin infusion in T1DM patients improved EPCs levels in a major extend that multiple daily injections, due to the lower glucose variability during the day [163]. In contrast, a recent study reported that the levels of some subtypes of EPCs (CD34^+^/CD133^+^/CD34^+^CD133^+^/CD34^+^KDR^+^) were reduced in newly diagnosed T2DM patients and did not vary after intensive insulin therapy; however, a pool of EPCs (CD34^+^KDR^+^CD133^+^) was increased in these patients and decreased after intensive insulin therapy, with a simultaneous reduction in oxidative stress and inflammation [164].

### Sulfonylureas

Sulfonylureas, drugs that stimulate the release of insulin reducing blood glucose, have also been studied regarding their effect on EPCs. For instance, an augmentation of EPCs levels (CD45^dim^CD34^+^ KDR^+^) was seen after 12 weeks of gliclazide treatment in T2DM patients, together with improved endothelial function and reduced levels of oxidant stress markers like serum malondialdehyde and superoxide dismutase [165]. However, glibenclamide did not improve EPCs levels (CD34^+^CD133^+^KDR^+^) in T2DM patients [166].

### Metformin

The effect of metformin, an oral hypoglycaemic drug derived from biguanide, has also been evaluated. Metformin administration to induced-T1DM mice decreased blood glucose levels, while increased EPCs numbers (Dil-acLDL^+^ FITC‐UEA‐I^+^ Sca-1^+^ Flk-1^+^) and improved in vivo wound healing and angiogenesis [167]. Also, metformin rescued the functionality of EPCs under HGE conditions through the AMPK/eNOS pathway [167]. Similarly, Han et al. [168] affirmed that metformin could improve EPCs levels (Sca-1^+^ Flk-1^+^) in an obese murine model of T2DM, rescued in vivo wound healing and in vitro angiogenesis, besides increasing NO production and reducing oxidative stress. At the clinical side, metformin increased cEPCs levels (CD45^dim^CD34^+^KDR^+^) in T1DM patients while reduced the number of circulating endothelial cells (cECs; CD45^dim^ CD133^−^ CD34^+^ CD144^+^ KDR^+^) and augmented the in vitro formation of colonies and the adhesion of pro-angiogenic cells to fibronectin [169].

Further, several clinical trials have shown that the combination of the above-mentioned drugs could be a plausible option to improve endothelial function in DM. Thus, the combination of gliclazide and metformin increased cEPCs levels (CD45^dim^CD34^+^KDR^+^) in T2DM patients more than metformin mono-treatment, although glucose control was similar in both groups [170]. Likewise, insulin plus metformin treatment augmented cEPCs levels (CD34^+^CD133^+^KDR^+^) and improved functionality in T2DM patients in a major extend that metformin mono-treatment [171].

### Thiazolidinediones

Regarding thiazolidinediones, a 12-week treatment of recently diagnosed T2DM patients with rosiglitazone reduced glucose levels, increased EPCs number (CD34^+^CD133^+^) and improved its migratory ability [172]. Besides, rosiglitazone treatment of EPCs (Dil-acLDL^+^ FITC‐UEA‐I^+^CD133^+^CD34^+^KDR^+^) isolated from healthy individuals, improved EPCs in vitro proliferation, migration and NO synthesis, while reduced apoptosis, restoring the negative AGEs-induced effects [173]. In addition, the administration of EPCS (Dil-acLDL^+^FITC‐UEA‐I^+^ CD31^+^vWF^+^KDR^+^CD14^+^) isolated from T2DM patients after a 2-weeks -treatment with rosiglitazone, promoted an improved in vivo revascularization in nude mice with carotid artery injury compared to EPCs isolated from T2DM patients before the treatment [174]. In an attempt to better understand the mechanisms by which rosiglitazone acts improving EPC function, Zhou et al. found that the 4-weeks rosiglitazone treatment of a T2DM murine model enhanced the in vivo wound healing and angiogenesis via stimulation of VEGF and SDF-1 [175]. Rosiglitazone also improved the in vitro EPCs migration and angiogenesis, and reduced IR signalling defects in EPCs [175].

Similarly, pioglitazone increased EPCs levels (Dil-acLDL^+^ FITC‐UEA‐I^+^ Vwf^+^ Tie-2^+^) and functionality while enhanced lipidic control in T2DM patients [176]. Likewise, the ex vivo administration of pioglitazone of early (Dil-acLDL^+^ FITC‐UEA‐I^+^ KDR^+^CD31^+^CD146^+^ vWF^+^CD45^+^CD14^+^) and late EPCs (Dil-acLDL^+^ FITC‐UEA‐I^+^ KDR^+^CD31^+^CD146^+^vWF^+^) isolated from individuals with impaired glucose tolerance, increased viability and their tube forming ability while reduced the expression of pro-inflammatory markers (ICAM-1, VCAM-1, TNF-α) [177]. Finally, an in vitro study reported that hyperglycaemia reduced the adhesion of EPCs (Dil-acLDL^+^ FITC‐UEA‐I^+^ KDR^+^CD31^+^) to arteries, and this effect was reversed by the pioglitazone treatment [178].

### DPP-4 inhibitors

The effects over EPCs of other treatments currently used to treat T2DM patients such as the dipeptidyl peptidase 4** (**DPP-4) inhibitors have been widely studied. For instance, a 4-week sitagliptin treatment increased EPCs (CD34^+^/CD34^+^KDR^+^) and SDF-1 levels in T2DM patients [179], in agreement with another study in which 12-weeks sitagliptin treatment doubled the number of EPCs (CD34^+^CXCR4^+^) in these patients [180]. In the same way, sitagliptin improved revascularization and angiogenesis in an T2DM murine model with HLI and could restore the detrimental effects of HGE in EPCs (CD34^+^KDR^+^), reducing the in vitro apoptosis and oxidative stress while increasing the tube formation ability and autophagy [181]. Besides, co-administration of sitagliptin and metformin promoted a major increment in EPCs number (CD34^+^KDR^+^/CD34^+^CD133^+^KDR^+^) compared with monotherapy, together with an increase of glucagon like peptide-1 (GLP-1), NO, and SDF-1α levels in T2DM patients [182]. Also, according to Nakamura et al. [183], sitagliptin promoted a major increase of EPCs levels (CD34^+^) rather than the alpha glucosidase inhibitor voglibose, although the endothelial function seemed to be similar in both groups. Similarly, Dei et al. [166] compared the effects of the DPP-4 inhibitor vildagliptin and the sulfonylurea glibenclamide, founding that although both controlled glucose levels, only vildagliptin achieved a significant augmentation in EPCs number (CD34^+^CD133^+^KDR^+^) with a reduction in SDF-1α levels. Further, Negro et al. [184] reported a similar increase in EPCs levels (CD133^+^KDR^+^CD45^−^/CD34^+^KDR^+^CD45^−^) after 4-months treatment with either DPP-4 inhibitor alogliptin and the sulfonylurea gliclazide. Also, a 12-weeks saxagliptin treatment of newly diagnosed T2DM patients improved endothelial function by increasing the flow-mediated vasodilation and increasing EPCs number (CD34^+^CD133^+^KDR^+^) in a similar manner as metformin treatment [185]. Then, Dore et al. [186] assessed the effect of combining saxagliptin and metformin treatment as compared to metformin monotherapy, noting that there were not changes in the CD34^+^ cEPCs number between both groups, although the number of CD31^+^ cECs increased in the combined group, together with a higher percentage of CD34^+^CXCR4^+^ in the CD34^+^ population, denoting an enhanced migratory ability of EPCs.

Interestingly, the administration of biocompatible membranes impregnated with saxagliptin boosted the in vitro EPCs migration and the in vivo diabetic wound healing in mice, while it augmented the expression of insulin-like growth factor I (IGF-1) and transforming growth factor-β1 (TGF-β1), compared to the membrane without drug [187]. In contrast to saxagliptin, linagliptin did not change the levels of EPCs in T2DM patients [188, 189], although it was associated with an increment in angiogenic T cells (Tang cells: CD3^+^CD31^+^CXCR4^+^) [189]. Finally, a recent study determined that the DPP-4 inhibitor teneligliptin increased (not significantly) the EPCs levels (CD34^+^) in T2DM patients after 28 weeks of treatment, although reduced SDF-1α levels, contrary to the tendency previously seen [190].

### GLP-1 receptor agonists

Other studies have evaluated the influence of GLP-1 receptor agonists (incretin mimetics) on EPCs. For instance, De Ciuceis et al. [191] compared the effects of exenatide and liraglutide, showing that only the first one was able to increase EPCs numbers (CD34^+^ KDR^+^) in T2DM patients after 4 and 7 weeks of treatment. The AngioSafe Type 2 Diabetes Study evaluated whether GLP-1 receptor agonists could be associated with the development of DR in T2DM patients, detecting no changes in the number of hematopoietic progenitor cells (HPCs: CD34^+^CD45^dim^) and HPCs cells with angiogenic activity (CD34^+^CD45^dim^CD133^+^CD31^+^) after the 4-weeks treatment with liraglutide [192]. These “negative results” supported the lack of association between GLP-1 Receptor Agonists and severe DR, given that a rapid change in EPCs number could be related to the development of the complication [192], in agreement with the LYDIA trial, that did not find any changes in the levels of CPCs (CD34^+^/CD34^+^ CD133^+^/CD34^+^ CD45^dim^/CD34^+^ CD45^dim^ CD133^+^) or EPCs (KDR co-expression with previous biomarkers), after 26-week liraglutide treatment, compared to sitagliptin [193]. Nevertheless, liraglutide improved the in vivo angiogenesis and recovered the blood supply in a murine model of T2DM with HLI, and recovered the in vitro hEPCs (CD144^+^CD34^+^VEGFR2^+^CD14^−^CD45^−^) migration and angiogenesis after HGE, being this related to the reduction of oxidative stress and over-expression of the human nuclear factor erythroid 2-related factor 2 (Nrf2) [194]. Besides, the 12-weeks treatment of dulaglutide in combination with metformin increased EPCs number (CD34^+^CD133^+^KDR^+^) and improved the in vitro EPCs proliferation, adhesion, migration, and tubule formation abilities in comparison with metformin monotherapy, in association with an anti-inflammatory activity and enhanced NO production [195].

### SGLT2 inhibitors

Sodium glucose cotransporter-2 (SGLT2) inhibitors have been associated with several effects on EPCs. Thus, the 16-week treatment with Canagliflozin in combination with metformin and/or insulin of T2DM patients increased EPCs (CD34^+^) expression of SDF1 and the migratory ability of EPCs in response to SDF1-α [196]. Canagliflozin did not affect EPCs number in comparison with placebo, although a reduction in EPCs number was seen at the beginning of the treatment that was reversed in the following weeks [196]. Similarly, Bonora et al. determined that after 12 weeks of treatment with dapagliflozin, the number of circulating stem cells CSC (CD34^+^) and EPCs (CD34^+^KDR^+^) were slightly reduced in T2DM patients, while the same duration treatment with empagliflozin diminished the CSC levels in a non-significant way [197]. However, a long dapagliflozin treatment (1.5 years) increased EPCs level [197].

### Statins and PCSK9 inhibitors

Apart from the treatments directly applied to tackle hyperglycaemia, DM patients usually receive additional drugs due to associated comorbidities that also can affect EPCs levels. For instance, hydroxy-methyl-glutaryl-Coenzyme A (HMG-CoA) reductase inhibitors, also called statins, are widely known to manage EPCs levels in hypercholesterolemic patients [198], and several studies have evaluated this pleiotropic outcome specifically in DM patients. Thus, atorvastatin significantly increased CPCs number (CD45^−^CD34^+^CD133^+^) after 8–10 weeks treatment in DM patients with CVDs, however, this increase was lower and non-significant in non-DM patients with CVDs [199]. In addition, although pitavastatin and atorvastatin reduced the lipidic profile in hyperlipidemic patients, only pitavastatin promoted a significant increase of EPCs (CD34^+^KDR^+^) [200]. Moreover, statin discontinuation increased the levels of different subpopulations of EPCs (CD34^+^KDR^+^/CD133^+^KDR^+^/CD34^+^CD133^+^KDR^+^/CD34^+^CD45^−^) as compared with a continuous treatment. The disruption of the treatment with statins caused a worse control of cholesterol concentration, and thus, the beneficial effects of the augmentation of EPCs could be reversed by the lipidic accumulation or even had a detrimental effect on PDR patients [201]. Further, Briguori et al. [202] evaluated the effect of the statin intensity on EPCs in DM patients with CAD who underwent drug-eluting stent implantation and were assigned high- or moderate-intensity atorvastatin therapy. Three months after the intervention, the number of EPCs (CD34^+^ KDR^+^ 133^+^/CD34^+^ KDR^+^) was higher and a reduction of restenosis was seen in the high intensity group compared to the moderate one. Further, statin treatment during 3 months before an AMI prevented the reduction of EPCs levels (CD45^dim^CD34^+^ KDR^+^ CXCR4^+^/CD45^dim^CD34^+^ KDR^+^ CD133^+^) that is usually caused after the incident, in both, DM and non-DM patients [203]. Moreover, a high intensity statin therapy after AMI also avoided the reduction of the EPCs levels [203]. Contrary, a high-to-moderate intensity statin therapy was associated with a reduction in the EPCs number (CD34^+^ KDR^+^), in comparison with a low intensity statin therapy in DM and non-DM patients with coronary angiography [204].

Apart from statins, the protease proprotein convertase subtilisin/kexin type 9 inhibitors (PCSK9i) constitute a promising lipid-lowering therapy currently used to attenuate atherosclerosis [205], whose effect over EPCs has also been assessed. Thus, Ben Zadok et al. [206] showed that the 3-months treatment with PCSK9i, reduced the lipidic levels and increased cEPCs number (CD34^+^ KDR^+^/CD133^+^KDR^+^) in CAD and PAD patients (10% of the patients suffered DM), and improved the in vitro colony-forming ability and viability of cEPCs. Interestingly, statins increased the levels of PCSK9 in both, T2DM and non-DM patients and, this increment of the PCSK9 levels after the statin treatment in the T2DM group was associated with a reduction of the total levels of EPCs (CD45^−^ CD34^+^) and eEPCs (CD45^−^CD34^+^CD146) [207]. These results disagree with most of the studies that associate the use of statins with the increment in EPCs [199–203], as mentioned above. However, the effect of statins on EPCs is somehow controversial, since results not only reflects an increment in EPCs, but also no change, or a reduction of its levels [199–204]. These conflicting results may be explained by the different statins employed or the intensity or duration of the therapy.

### Renin-angiotensin system (RAS) inhibitors

Angiotensin II receptor antagonists, usually employed to reduce high blood pressure, have been recognized to increase the EPCs levels in DM patients. Indeed, the antagonists Olmesartan and Irbesartan were found to increase the levels of EPCs (Dil-acLDL^+^ FITC‐UEA‐I^+^) in T2DM patients after 4 and 12 weeks of treatment [208], while the angiotensin-2 receptor blocker valsartan increased the levels of CD14^+^ KDR^+^ cells in DM patients with asymptomatic CAD [209]. Also, the oral administration of perindopril increased the number of cEPCs (CD34^+^CD133^+^KDR^+^) in T2DM patients that suffered AMI, at days 1, 3, 5, 7, 14, and 28 after percutaneous coronary intervention [210]. The treatment also increased the plasma levels of VEGF and SDF-1α, reduced the high-sensitivity C reactive protein (hsCRP) levels and improved the clinical outcomes of the patients [210]. Similarly, the renin inhibitor aliskiren, but not hydrochlorothiazide, improved the vascular function T2DM-hypertensive patients through the reduction of the blood pressure, the increase of the brachial artery flow-mediated dilatation, and the enhancement of the left ventricular function, and it also promoted the augmentation of EPCs (Dil-acLDL^+^ FITC‐UEA‐I^+^) levels and the pool of CD34^+^CD133^+^ cells [211].

### Other treatments

Apart from the above-mentioned treatments, other drugs applied in DM patients have also been demonstrated to influence EPCs number and functionality. For instance, calcium channel blockers (CCBs), drugs mainly employed to treat hypertension [212], have been shown positive effects over EPCs, by improving EPCs functionality as well as increasing the number of EPCs in several in vitro studies [213, 214]. Similarly, Alpha-glucosidase inhibitors (AGI), commonly used to reduce glucose levels in T2DM [215], were found to increase the levels of EPCs (Sca-1^+^ Flk-1^+^) in a T2DM murine model, and improved the in vivo wound healing and angiogenesis through the Akt/eNOS signalling pathway [216]. In the same line, Jeong et al. compared the action of the 10-weeks-treatment with two antiplatelet drugs, the P2Y_12_ receptor antagonists ticagrelor and prasugrel, assessing that only the first one was able to increase the level of EPCs (CD34^+^KDR^+^/CD34^+^CD117^+^/CD34^+^CD133^+^) in T2DM patients with non–ST-segment elevation acute coronary syndrome. This increase was associated with the higher serum levels of adenosine caused as a pleiotropic effect of ticagrelor [217].

Finally, a combination of different treatments may be useful in the management of the vascular health in DM. In this sense, a multifactorial treatment including mono-, dual-, triple- or quadruple therapies with metformin, aspirin, statins and angiotensin II blockers increased the EPCs levels (Dil-acLDL^+^ FITC‐UEA‐I^+^) of T2DM patients after 90 days, especially when applying the quadruple therapy [218].

## Conclusions

The enormous number of studies highlighted here corroborate the potential of using EPCs as biomarkers of DM and its vascular-related complications, given the effect that these pathologies exert over EPCs number and function. Nevertheless, the variability seen between the studies might be explained by the lack of consensus in the surface markers employed to define these cells, but also on the influence that DM comorbidities can exert over these cells. Additionally, the treatments applied to DM patients also affect the number as well as the functionality of these cells. Finally, apart from the biomarker role assigned to these cells, future studies should further evaluate the molecular mechanisms by which EPCs role is impaired in DM and moreover, in the presence of vascular diabetic complications, in order to provide more specific and personalized therapies to diabetic patients.

## Data Availability

Not applicable.

## Abbreviations

AGEs: Advanced glycation-end products
AGI: Alpha-glucosidase inhibitors
AMI: Acute myocardial infarction
BID: *Bis in die*
BM: Bone marrow
CACs: Circulating angiogenesis cells
CAD: Coronary artery disease
CathB: Cathepsin B
CCBs: Calcium channel blockers
CECs: Circulating endothelial cells
cEPCs: Circulating endothelial progenitor cells
CFU: Colony-forming units
CLI: Critical limb ischemia
CPCs: Circulating progenitor cells
CRD: Chronic renal disease
CSCs: Circulating stem cells
CVDs: Cardiovascular diseases
Dil-acLDL: 1,1‐Dioctadecyl‐3,3,3,3‐tetramethylindocarbocyanine‐labelled acetylated low‐density lipoprotein
DM: Diabetes mellitus
DN: Diabetic nephropathy
DNeu: Diabetic neuropathy
DPP-4: Dipeptidyl peptidase 4
DR: Diabetic retinopathy
DVCs: Diabetic vascular diseases
ECFCs: Endothelial colony-forming cells
ECs: Endothelial cells
eEPCs: Early endothelial progenitor cells
eNOS: Endothelial nitric oxide synthase
EPCs: Endothelial progenitor cells
EPO: Erythropoietin
EPOR: Erythropoietin receptor
FITC-UEA-I: Fluorescein‐isothiocyanate (FITC)‐conjugated Ulex europaeus agglutinin lectin
GDM: Gestational diabetes mellitus
GLP-1: Glucagon like peptide-1
GSK3β: Glycogen synthase kinase-3β
HbA1c: Glycosylated haemoglobin
HC: Healthy control
HGE: High glucose exposure
HIF-1: Hypoxia inducible factor 1
HLI: Hind limb ischemia
HMG-CoA: Hydroxy-methyl-glutaryl-Coenzyme A
HPCs: Hematopoietic progenitor cells:
hsCRP: High-sensitivity C reactive protein
hEPCs: Human endothelial progenitor cells
IGF-1: Insulin-like growth factor I
IR: Insulin resistance
LEA: Lower extremity amputation
NO: Nitric oxide
NPDP: Non-proliferative diabetic retinopathy
PAD: Peripheral artery disease
PB: Peripheral blood
PBMCs: Peripheral blood mononuclear cells
PCs: Progenitor cells
PCI: Percutaneous coronary intervention
PCSK9i: Protease proprotein convertase subtilisin/kexin type 9 inhibitors
PDGF: Platelet-derived growth factor
PDR: Proliferative diabetic retinopathy
pEPCs: Putative endothelial progenitor cells
PPARα: Peroxisome proliferators-activated receptor α
RAS: Renin-angiotensin system
ROS: Reactive oxygen species
SGLT2: Sodium glucose cotransporter-2
SMPCs: Smooth muscle progenitor cells
STZ: Streptozotocin
T1DM: *Type 1 diabetes mellitus*
T2DM: *Type 2 diabetes mellitus*
TGF- β1: Transforming growth factor-β1
TID: Tir* in die*
VEGFR-2: Vascular endothelial growth factor
VLDL: Very low-density lipoprotein
vWF: Von Willebrand factor
